# Self-management perspectives of elderly patients with multimorbidity and practitioners - status, challenges and further support needed?

**DOI:** 10.1186/s12875-021-01584-9

**Published:** 2021-11-26

**Authors:** Amanda Breckner, Catharina Roth, Katharina Glassen, Michel Wensing

**Affiliations:** Department of General Practice and Health Services Research, Heidelberg University Hospital, Marsilius Arcades, West Tower, Im Neuenheimer Feld 130.3, 69120 Heidelberg, Germany

**Keywords:** Self-management, Self-management support, Multimorbidity, Social participation

## Abstract

**Background:**

Patients with multimorbidity (here defined as three or more chronic conditions) require constant treatment and care. Furthermore, they have to manage their health and diseases in daily life. Offering support to patients’ medical self-management is an important task of primary care. The aim of this study was to explore, what further support is needed from the perspective of patients’ and primary care practitioners.

**Methods:**

A qualitative study using individual semi-structed interviews with 17 patients with multimorbidity and 7 practitioners (4 primary care physicians and 3 practice assistants) was conducted in Germany. Data were audio-recorded, pseudonymised and transcribed verbatim. Data analysis was performed using qualitative content analysis to structure data into themes and subthemes. All data were managed and organised in MAXQDA.

**Results:**

The three broad themes: current status, challenges and further support emerged. Patients reported on unfulfilled needs regarding role or emotional management, like coping with loneliness, loss of independence and, changing habits. The importance of social contact was highlighted by patients and practitioners. Patients articulated further support from their primary care practitioners on coping with the disease. Practitioners’ wished for further support in aspects of social participation, public transport, and community resources.

**Conclusion:**

Challenges regarding self-management of elderly patients with multimorbidity may be addressed by harnessing social support and community initiatives.

## What this study adds

- Support in primary care practices is mainly medical self-management support, whereas patients wished for more emotional support.
- Including a social medical service in municipality or combining social events and medical lectures could address challenges of elderly patients.
- Cooperation between primary care practices and social initiatives may activate elderly people.

## Background

Worldwide multimorbidity remains a significant challenge in healthcare systems. In Germany from 2006 to 2015, the prevalence of multimorbidity increased from 34.2 to 44.6% in this large representative sample. The same study found an increase of prevalence of multimorbidity in Europe in this 10 year period from 38.2 to 41.5% [1]. In our study, multimorbidity is defined as the coexistence of three or more chronic conditions in an individual. The criterion of three or more chronic conditions was chosen to include patients for whom the diseases represent a certain burden [2]. Multimorbidity is associated with decreased quality of life, functional decline and increased health care use [3]. Patients with multimorbidity have to face not only their chronic conditions but also, their health consequences as well as their day-to-day activities such as taking medicine, recording disease symptoms or coping with the situation. Thus, chronic diseases require constant attention of the affected individuals. Therefore, people with multimorbidity often have to develop effective skills to manage activities of daily living, their health and disease. Self-management has been defined as “the practice of activities that individuals initiate and perform on their own behalf in maintaining life, health, and well-being” [4]. In this context Loring and Holman identified a theoretical model of three self-management tasks: medical management, emotional management, role (social) management as well as six specific skills: problem solving, decision making, resource utilization, the formation of a patient-provider partnership, action planning, and self-tailoring [5].

The interest in self-management approaches and interventions for people with multiple chronic conditions as well as factors affecting their self-management has increased in the last years. A review by Gobeil-Lavoie et al. identified challenges to self-management for patients with complex healthcare needs [6]. They pointed out that the patients may have to prioritise care based on one dominant disease. Although, they have a great opportunity to use personal experiences acquired in the past to improve their self-management skills, depression, psychological distress, low self-efficacy as well as receiving conflicting information from healthcare providers poses an increased risk [6]. However, in a review by Poitras et al. on effective elements in patient-centred and multimorbidity care, self-management support interventions by different healthcare providers were found to result in positive impact for patients with chronic diseases on e.g. patient satisfaction and quality of life [7]. Thus, primary care physicians and their practice team play an important role in the care of people with multimorbidity [7]. Moreover, Freilich et al. found that health care professionals could collaborate with patients to individualise self-management support through personal continuity and patient-centred consultations [8].

Individualization could also mean that a patient decides to leave his/her physician in control or not to engage in a healthful behaviour, which also reflects a management style [5]. This could be the case for elderly patients in particular, whereas it has been shown that activation decreases [9]. The longer patients live with chronic diseases, the more likely they may get used to everyday treatment requirements, such as taking medication regularly and monitoring symptoms. Furthermore, a study from Kristensen et al. demonstrated that multimorbidity is on the one hand associated with increased loneliness and social exclusion but on the other hand with an increased network size, which may be related to the increased need for support [10]. This may indicate that self-management, particularly for the elderly generation with multimorbidity, is connected to different aspects of social relationship. Previous studies have shown that many elderly people are not satisfied with their social relationships and their social participation [11, 12]. About 18% of the patients felt often or occasionally lonely, but rarely shared this issue with their physician [11]. Another study showed that more than a half of the patient sample wants to discuss loneliness or social activities with their primary care provider [12]. Moreover, Loring and Holmann reported that most health promotion do not deal systematically with the self-management tasks of role and emotional management [5, 7].

Furthermore, the two reviews from Poitras et al. [7] and Gobeil-Lavoie et al. [6], respectively identified different aspects where future research is needed. Poitras et al. declared that more research is needed on elements of patient-centred and multimorbidity care, such as a long-term management plan or engaging the patient as a partner [7]. Gobeil-Lavoie et al. pointed out that more studies should empirically validate the characteristics of self-management by patients with complex health needs and could contribute to the experience of these patients [6].

In summary, more evidence is needed about what support elderly patients with multimorbidity need from their perspective and from the perspective of primary care physicians and their team (practitioners). We therefore conducted a qualitative interview study to explore the needs for support of self-management in patients with multiple chronic health conditions.

## Methods

### Study design

We conducted a qualitative study with open-end questions for focused interviews on self-management and self-management support with randomly chosen patients and practitioners.

### Study setting

This qualitative study was an additional part of a mixed-methods study named "Development and Validation of Quality Indicators for Multimorbidity" (MULTIqual), which aimed at developing a set of quality indicators for primary care providers for patients with multimorbidity [13] (Schulze J, Glassen K, Pohontsch N, Blozik E, Eißing T, Höflich C, Breckner A, Rakebrandt A, Schäfer I, Szecsenyi J, Scherer M, Luehmann D: Measuring the quality of care for older adults with multimorbidity: Results of the MULTIqual Project, In Review at The Gerontologist). The qualitative part of the larger study included 47 patients with multimorbidity and the cross-sectional survey study included 346 patients with multimorbidity in primary care practices in two German regions (Hamburg and Heidelberg). Patients were selected on the presence of three or more documented chronic conditions.

For this study patients and practitioners from the study site of Heidelberg and surroundings were invited. Heidelberg is a city with around 160.000 inhabitants in the southwest of Germany. The surrounding districts have about 548.000 inhabitants.

### Sampling and recruitment

For this qualitative study 25 patients were randomly selected with a random generator and invited to take part in a semi-structured interview. Furthermore, all 18 primary care physicians as well as 5 practice assistants from the study site Heidelberg, participating in the MULTIqual project were invited to take part in an interview. All patients were treated in one of the medical practices of the invited physicians.

### Data collection

First, the interview guideline for the interviews with the patients was developed. Questions were open-ended and about 1) how patients perceive their self-management and their multimorbidity at that time, and 2) support they receive by their primary care practice as well as support they may wish for. Corresponding open-end questions regarding self-management of patients with multimorbidity and the support they receive by their primary care practice were developed for the interviews with practitioners. Table 1 presents both interview guidelines.

**Table 1:** Questions in the interview guidelines

Questions for patients	Questions for practitioners
**Current status - Perception of self-management**	
a. How do you get involved in decisions around your diseases/health?	a. How do patients get involved in decisions about their diseases/health?
b. What do you do to improve/maintain your health (at home)?	b. How do patients manage their multiple conditions from your perspective?
**Current status – Self-management Support**	
d1. How does your primary care practice support you regarding self-management of your diseases?	d. How do you support patients with multimorbidity in your practice?
d2. How does your primary care practice motivate you to do improve/maintain your health?	b1. How do you motivate patients for self-management? b2. How do you motivate patients to participate in patient training programmes?
**Challenges**	
c. What disease is most stressful to you? Do you also get more involved within this disease?	c. How do you think patients perceive their disease? Is one disease in the foreground?
What problems do/did you face in the context of your disease? Is there anything that would make it easier for you to cope with your situation?	Which problems do patients with multimorbidity often have to handle?
**Further support**	
e. What further support would you like to receive regarding self-management of your chronic conditions?	e. Which further support possibilities for people with multimorbidity do you know? Which support do you would wish for your patients?
f. Is there anything else concerning your self-management you want to tell me?	f. Is there anything else concerning the self-management of patients with multimorbidity you want to tell me?

Individual semi-structured interviews were conducted with patients and practitioners (primary care physicians and practice assistants) by the first author AB with a background in health services research. All patient interviews, but one that was conducted via telephone, were conducted in their own homes between December 2019 and March 2020. All practitioners were interviewed via telephone between May 2020 and July 2020. The average length of interviews were 19 min (range 7–54). All interviews were digitally recorded with consent and transcribed verbatim.

### Data analysis

We used an inductive-deductive qualitative approach to content analysis to structure all interviews into themes and subthemes [14]. Firstly, transcripts were read thoroughly by the authors AB and CR (both background in health services research) to identify key themes. A preliminary system of themes was developed deductively from the interview guide and inductively from additional content of the interviews. Then, all interviews were coded line by line to structure data into themes and subthemes by AB and CR. Analyses were compared and discussed by the two researchers. Coded themes were modified when applicable. Furthermore, the system of themes and codes were discussed with KG (background in primary care medicine) to ensure validity. All data were managed and analysed using MAXQDA. Quotes presented in this article were translated from German into English.

### Ethics approval and consent to participate

Ethical approval was obtained of the Medical Ethics Committee of the Medical Faculty of Heidelberg University (S-665/2018) prior to the start of the study. All study participants gave written informed consent prior to their participation in the study. Research conducted in this study was performed in accordance with the Declaration of Helsinki.

## Results

### Demography

Of the 25 patients with multimorbidity invited, 17 took part in an interview. During one patient interview the spouse of the patient was present. Of the 22 practitioners invited, 7 practitioners (4 primary care physicians and 3 practice assistants with further training) participated in an interview.

Tables 2 and 3 show the characteristics of participants. Patients (7 men and 10 women) were on average 76.5 years old with the youngest patient being 66 and the oldest patient being 89. The mean self-reported chronic diseases were 8 (Range 4–15) whereas they had a mean of 13 diagnoses reported from their primary care physicians. The most frequent diseases were chronic heart failure, hypertension, arthrosis, and metabolic disorder. Their educational qualification was relatively low with 41.8% of the sample being in Level 1 of the Comparative Analysis of Social Mobility in Industrial Nations (CASMIN), including patients with inadequately completed general education or basic vocational qualification only and 41.8% in CASMIN Level 2, including patients with a medium education. Income varied between 1100 € and 5000 € with most patients having an income between 1500 and 2000 €. All patients were retired, only one was working in part time. Seven patients lived in urban areas, whereas 10 patients lived in rural areas. Only one patient visited a patient education and a self-help group whereas 3 patients received a self-management plan .

**Table 2:** Demographics of patients

Patient	Age	Gender	Casmin Level	Income	Marital Status	Residence
1	79	Male	1	Between 1500 and 2000 €	Divorced	rural
2	83	Male	3	Between 2000 and 2500 €	Widowed	rural
3	78	Male	1	Between 3500 and 4000 €	Married	urban
4	75	Male	1	Between 3000 and 3500 €	Married	urban
5	78	Female	2	Between 2000 and 2500 €	Widowed	rural
6	66	Female	1	Between 1500 and 2000 €	Married	rural
7	66	Female	2	Between 4000 and 5000 €	Single	rural
8	89	Male	2	Between 2500 and 3000 €	Widowed	urban
9	68	Female	2	Between 350 and 4000 €	Divorced	urban
10	85	Female	2	Between 1100 and 1300 €	Widowed	rural
11	79	Female	1	Between 1500 and 2000 €	Widowed	rural
12	82	Male	3	Between 4000 and 5000 €	Married	rural
13	72	Male	2	Between 3500 and 4000 €	Married	rural
14	70	Female	1	Between 2000 and 2500 €	Widowed	urban
15	78	Female	2	Cannot tell	Widowed	rural
16	84	Female	1	Between 1500 and 2000 €	Divorced	urban
17	70	Female	3	Between 3000 and 3500 €	Divorced	urban

**Table 3:** Demographics of practitioners

Practitioner	Role	Age	Gender	Number of years qualified
1	Practice Assistant, VERAH (Care Assistants in General Practice (Versorgungsassistentin in der Hausarztpraxis, VERAH))	55	Female	40
2	Practice Assistant, VERAH	43	Female	11
3	Practice Assistant, Case Manager	46	Female	26
4	Primary care physician	49	Female	19
5	Primary care physician	63	Male	23
6	Primary care physician	59	Female	14
7	Primary care physician	58	Female	27

Practitioners (7 women, 1 man) were on average 53 years old with the youngest practitioner being 44 an the oldest being 63. They reported on average about 23 years of professional experiences (Table 3).

### Overview

The analysis highlighted three main themes regarding self-management of patients with multimorbidity from patient’s and practitioner’s view: (1) current status – what do patients do for their health, which support is available in primary care practices; (2) challenges that patients with multimorbidity and practitioners face; (3) Need for further support. All themes included subthemes and are presented in Table 4.

**Table 4:** Main themes and categories

Theme	Category	Subcategory
Current status	Self-management tasks	Nutrition/ Diet
		Training
		Medication
		Information/ Keeping up-to date
		Social exchange
		Responsibility
		Coping with multimorbidity
	Self-management support	Motivation
		Recommendations
		Exchange with physicians and practice assistants
		Rehabilitation services
		Organisational aspects
Challenges	Multimorbidity and its challenges	Pain
		Loneliness/ lack of social networks
		Loss of independence
		Changing habits/ lifestyle change
		Treatment burden
		Mobility restrictions
		Being a burden for family/friends
	Challenges for practitioners	Allocation of time and resources
		Strategies for motivation
Further support	Concerns expressed from patients	Support for specific problems
		Support for general problems
	Concerns expressed from practitioners	Public transport
		Support of relatives
		Government support
		Social medicine

#### Current status – what do patients do for their health, which support is available in primary care practices

##### Self-management tasks

Patients declared a various number of self-management tasks ranging from taking their medicine and doctor’s visits over changing diet or keeping proper diet to doing exercises. Furthermore, they stated that they have to keep up to date, inform themselves about their diseases as well as document disease-relating data like diabetes or blood pressure values.

Another crucial part for patients with multimorbidity were the exchange and discussions with relatives or reference persons about their chronic diseases and their treatment. Moreover, many patients declared how important social contact is for their well-being. They reported about sport courses, choirs or community events where they go to meet other people. Some also talked about carpools they arranged to take other elderly people with them to get to public events.

“I live more or less alone and I would like actually then when I do something, I want to do it in company, that's why I'm looking for all the courses. I also do as I said yoga and, in the gym, you also get to know new commonalities with any other people, you can talk, there you want, yes, also a bit of social contact!” (Patient 9)

Almost all patients saw themselves responsible for their own health, but at the same time they articulated how important it was to trust and keep in touch with their primary care physicians.

“I don't need motivation, I'll do it myself! You see... I say to myself, ”This is my life so I have to do something about it!“ No? I should move around and do the other... That's just the way life is, isn't it?” (Patient 12)

Furthermore, most patients felt that they were coping well with their diseases and tried to integrate medical advices into their life. There were differences in the time spent dealing with the disease. Some said that it is best not to think about the disease too often whereas others focused and align their lives according to the diseases.

“I live my life and try to listen to medical advices regarding what I need to do that I can be a... A... I'll say live an acceptable life. So, no runaways! But with me there were outliers, no doubt, but today I live according to the medical guidelines! But not spartan! Everything in a reasonable, compatible measure.” (Patient 8)

##### Self-management support

Patients and Practitioners said that self-management support should be individualised and varies from patient to patient. Most patients reported that they are satisfied with the motivation and support they get from their physicians and the practice team.

“I am already [motivated] by my primary care doctor. He says when something is not right. He doesn't mince his words.” (Patient 1)

They said that they can talk and ask their physicians and practice teams what they want and discuss for example issues that are not necessarily disease-related such as loneliness, stress or anxiety. Recommendations were often not implemented on a regular basis. Most patients knew that the recommendations could help but apply them only in the case of pain or symptomatic deterioration.

“Yes, well, I mean, if I... I also had breast cancer, so I still go to cancer follow-up care, I go regularly do cancer screening and still go every quarter of a year. I also insist on not going every six months or yearly to follow-up care and they all know me well. (…) And if I have a question or if something is wrong, then I talk about it briefly, then I get an appropriate answer and then it's good. So if there's something I can't deal with, then I ask and that's enough for me.” (Patient 16)

Rehabilitation services in specialised in-patient clinics with offers of e.g. physiotherapy or occupational therapy were perceived as very helpful by the patients. They learn much about which training or which diet fits best to their diseases, especially at the onset of the disease. Whereas patient education was only visited by two participating patients and was not mentioned by the patients when asking for self-management support. However, some patients explained that they visit a rehabilitation at their own charge regularly.

“I was in a rehabilitation for six weeks and learned a lot of helpful things there about how to deal with..., so initially I had a lot of problems getting my everyday life going again, because when you have two-thirds of your lungs removed, it's a bit of a difficiult, I'll say that now.” (Patient 9)

Furthermore they declared how important participation in decision-making reagarding medical care with their primary care physician is.

“Of course, it is also important that I know where I have to go, to which doctor. And I have the advantage with my primary care doctor that I can also discuss this with him. And that I also get the referrals to the specialists from him. That works relatively well. Of course, he also has my data in the computer and can see exactly what is good for me. That is a big advantage and I can take advantage of it.” (Patient 5 )

Practitioners reported that they stay in regular contact with their patients with multimorbidity. Support in primary care practices is available mainly via consultations, telephone calls and regular check-ups.

“Yes, in principle, essentially through offers, conversations... Proactive questions and offers to talk about problems and find a solution together. So, the patients are very different, some would not say anything of their own, you have to be proactive.” (Physician 1)

The practice assistants highlighted that they support patients also in organizational aspects like filling out an application for e.g., a rehabilitation.

“Yes, often the patients come when they have been approved for medical rehabilitation, then they have several pages, they have to fill out themselves. Often, I fill it out together with them, because often the questions are not understood at all, because they are written in a different language that some patients really do not understand, especially in the medical nomenclature.” (Practice assistant 2)

Practitioners articulated that it is crucial to address patients’ needs and concerns. However, adhering to the needs-based recommendations as well as participation depends on the individual patient. Just like the patients, practitioners told that in case of pain patients are highly motivated to be involved in their care.

“It always depends on the... On the individual patient, one is more motivated and the other a bit less... You often get into conversation when you take blood samples, you know the patients for some years and then you find out a little bit, the sensitivity how to motivate one or the other a little bit.... Yes, to motivate them by getting into conversation with them.” (Practice asisstant 2)

#### Challenges that patients and practitioners face

##### Multimorbidity and its challenges

Patients and Practitioners stated that there are mostly one or two diseases in the foreground. Mainly the one who is causing pain or direct negative effects for the patient, e.g. incontinence.

“Yes, well, it's the pain that weighs on me the most, the consequences of what can still come of it, I have to say honestly, I don't think too much about that now, I just try to move and I think to drag the whole thing out.” (Patient 7)

When participants were inquired about the challenges they face or experience with multimorbidity, patients talked about various aspects like loneliness, loss of independence, changing habits as well as handling pain and treatment/ disease burden.

“I used to work in the [social institution] and now I'm a pensioner and if I don't go out myself, it's sometimes hard for me to be here alone in my half of the house. So I need the strength, the spiritual strength to go out and I also need that to keep myself mobile and I hope that will last a little longer. That I can continue to be mobile a little bit.” (Patient 5)

Practitioners also perceived various challenges of their patients with multimorbidity. Most practitioners indicated problems of mobility restrictions due to limited mobility or lack of public transport. If there are no ancestors or reference persons, this problem as well as social support and organizational supports is exacerbated.

“They usually travel by public transport and are on the road for hours. I have a patient who told me that it actually took 4 hours to get from the university clinic to the eye clinic, from back and there... Return journey and outward journey, right? 4 hours is an enormous burden for an almost 80-year-old woman during the day.” (Practice assistant 2)

In addition, some patients do not want to be a burden for their family and friends or for their physician and their practice team.

“There are patients who express themselves little and also tell little about their problems, it is simply not to be a burden to others.” (Practice assistant 2)

Another problem for patients with multimorbidity perceived by the practitioners are aspects of lifestyle changes like changing of diet, quieting smoking or doing sports to cope with the diseases.

“A change of diet is of course very difficult - everyone knows that... Quitting smoking is also very, very difficult. But we always try to reflect this in the patient, that he is his own therapist, that we only support him with medication and so on, he actually has to lead his own life and must, for example, somehow find a form of exercise that he enjoys, yes?” (Physician 4)

An wide ranging problem from the point of view of practitioners is the lack of a social network with friends or family, which affects many elderly people. This exacerbates the problems described above.

“I think it depends on the personality of the people, how they have otherwise managed themselves in life and of course also in the social context, for example having a very good network of relatives and so on, that is of course also a difference to those who are single, but there it is also very different.” (Physician 1)

##### Challenges for practitioners

Practitioners often wanted to help to address the problems of their patients which causes also challenges for themselves. One challenge for the practitioners is the allocation of time and resources. Patients with multimorbidity and their treatment is highly time consuming if done adequately. One physician mentioned that he did not find the right method to motivate patients with multimorbidity to self-management or lifestyle changes which is difficult to accept for him, too.

“I also often ask myself how much of what we talk about here.... And I really take a lot of time and like to listen for a long time and try to explain certain contexts but how much ultimately sticks or is taken away, yes? Because they then present themselves again a few months later and we have the same topic on the table again as it was before, without anything having been changed in part, yes? [...] I mean, we make every effort, we are open to everything, but it is difficult, yes? It's really a difficult undertaking!” (Physician 2)

#### Need for further support

##### Concerns expressed from patients

Although patients mostly stated they have good medical care and do not need further support, some declared different wishes. On the one hand, some patients expressed precise demands like regular stays in rehabilitation clinics, more financial support and opportunities from their health insurances, the regular exchanges between their attending physicians as well as more support regarding for example incontinence.

“Yes, it would actually be good if rehabilitation could take place regularly every four or five years for some chronic diseases, such as fibromyalgia, even if it is only an outpatient rehabilitation. That would do a lot of good. If it were simply normal that you could do it every few years.” (Patient 14)

“Yes, bladder weakness, how to deal with it. I wonder if there's anything newer. Several people probably have it and maybe that's it, I don't know. Nobody can help me with that, it's just all that... this bladder weakness is already ugly.” (Patient 15)

On the other hand, patients expressed concerns regarding general problems and challenges occurring in multimorbidity and old age. They wished for support and consultation regarding stress or coordination and organisation in difficult times as well as more exchange at the beginning of the disease. Moreover, patients wished for consultation of additional offers or things they can do at home.

“Yes, precisely the age-appropriate thing. Movement, age, being alone. These are the factors that I have to deal with.” (Patient 5)

“So, what possibilities there will be, what you could do in addition.” (Patient 6)

##### Concerns expressed from practitioners

Practitioners on the other side with a more comprehensive view suggested also various wishes and demands. Practitioners declared that mobility in case of public transport as well as costs involved could be improved especially in rural areas. In rural areas, it may be the case that patients have to get to the nearest town for specialist care which often involves using different types of public transport. Moreover, the application for using a taxi as well as to get remunerated it from the health insurance should be facilitated as well, as it is often difficult for elderly people to handle. Some practitioners reported that they see great need for support, especially for those patients living alone. Not uncommon, the practice assistants have to help to organise a driving service or have to involve a nursing service.

“Yes, so what comes to my mind spontaneously is actually to improve mobility somehow, that is, through more driving services or however you do it, because that is often a hurdle. And then also for people for financial reasons or something. I mean, sure, if you can afford it, you can take a taxi, but it's just not the case that they can or want to do that... And then it is often difficult. Because it is often a cost issue. And then, of course, depending on the pension office, you can apply for some kind of handicap, degree of severe handicap and characteristic mark, which is often a bit cumbersome, because it also requires some teamwork, so it's a bit more complicated and complex, I would sometimes like it to be a bit easier, that you can simply issue it for old people... A taxi voucher, regardless of whether they have a special sign or not, if you need it for any necessary journeys, of course.” (Physician 1)

Another aspect practitioners raised was the support of the relatives. At the beginning of a disease the management is often difficult for the patient and his/her relatives. More and specific information about what is needed to care for the patient at home could benefit both. The basic idea that the patient and his/her environment should be involved in the chronic disease and its care should be promoted.

“I just see the problem that self-management has to be learned first. To know, “Where do I start?” I would very much like the younger ones, the children, the daughters, the sons, to be the first to (be involved). So many things could be done if the children were not overwhelmed with the situation of their parents, because they have too much by themselves. They could do a lot more at home, I would say, in their familiar environment, independently, if they were better informed. ” (Practice assistant 3)

Furthermore, practitioners wished for more government support like taxes on sugar, more offers in giving up smoking and more sport programmes in companies and schools. General things which could help preventing chronic diseases. In addition, practitioners explained that chronic disease should be uncoupled from debt and connected to more positive impulses. One example was the connection of lectures and patient education with a joint meal whereas people can meet each other. This could be an event for elderly people to contact others and learn something about the management of their diseases in a casual atmosphere.

“When I think of my mother, who lives in a village with 1400 inhabitants, if the old people's meal were to take place, let's say, in the community centre and they had to go there every day at noon, that would be much better for the old people than the Malteser or whoever else sheds a Styrofoam box in front of their door. And they would have to walk there and eat there and then you can have a coffee and then you go home again and then it’s a non-binding social contact where you also happen to have eaten. And then you can attach to such a contact, for example, a little lecture on “How much cheese should I eat if I have high fats.“. ” (Physician 3)

One physician suggested the need of more interest in social medicine in the daily work of primary care and in communities. The wishes mentioned are in line with this topic.

“The essential topic is a topic in which we are not trained, that we do not get paid for and that we still have to take care of every day, and that is social medicine. So how do people manage their lives and has anyone ever asked about that. Now, in the Corona telephone survey, we have gained more insight into the patient's everyday life than usual, because if he cannot come to us and we telephone him in his home environment, so to speak, then we can also ask: You live on the third floor, how do you actually get up and down, who did the shopping for you, who cleans, who makes sure that you take your medication regularly? You know, we are then confronted with the derailment, the relatives who say that everything is not working out at all and who is organising it now and that is not my job at all, I am not paid for it and it is not my job to organise it but it ends up with me, because I say it now because the state is not doing its job. Every municipality should have a social medical service where relatives can turn to when it no longer works with the medication, when it no longer works with the cooking, when it no longer works with the cleaning. And there are almost always medical reasons why it doesn't work anymore.” (Physician 3)

## Discussion

### Principal findings

This qualitative study explored the perspectives of elderly patients with multimorbidity and their practitioners about the current status of self-management, their perceived challenges and the need of further support. Our findings highlighted that medical management is present in the day-to-day activities of patients with multimorbidity as well as in their care, whereas the emotional and role management is mostly missing. Challenges perceived by the patients were mainly loneliness, loss of independence, changing habits as well as handling pain and treatment/ disease burden. Thus, aspects of creating new meaningful behaviour or life roles, where support may be needed. Challenges for the patients, perceived by the practitioners are lifestyle changes, as well as in some cases lack of social networks. Challenges for practitioners themselves were mainly the allocation of time and resources. Asking for further support concerning self-management, patients mainly named aspects and needs to talk with their primary care physician. Whereas practitioners expressed mainly needs in the public sector, such as accessible public transport, more community events for elderly people where they can gather for a meal and socialise, a social medical service in each municipality, as well as more preventive measures from the government and more extensive involvement of relatives.

### Challenges that patients and practitioners face

A study from Rogers et al. in the United Kingdom highlighted that primary care professionals contribute mostly to illness specific work e.g., interpreting measurements or understanding symptoms and less in emotional work e.g., comforting when worried or anxious, everyday matters including well-being and companionship. In the study from Rogers et al. the support network of people with long-term conditions consists to 15,5% of primary care professionalsand patients did not expect primary care physicians to mediate their self-management support [15]. In contrast patients in our study shared emotional problems with their primary care physicians and their team and wished also for more support. Additionally, practitioners declared that they spend much time talking with their patients with multimorbidity and their problems but also highlighted the need for more public and government support. Like the practitioners in our study, Rogers et al. [15] highlighted the need of open system resources in domestic and community settings accessible to people with long-term conditions. However, the authors pointed out this need due to the low involvement of primary care professionals in self-management support in their sample. The background of the differences to the expectations of our patients remains unclear, as UK’s and Germany’s healthcare are not quite different concerning psychosocial inclusion in medical training. In Germany, the primary care physicians remain the first contact for elderly patients with multimorbidity and often community resources like sport courses are conveyed via the primary care practices.

From the patient perspective challenges occurred mainly in role and emotional management like loneliness, loss of independence, changing habits or handling pain and treatment/ disease burden. Our results concerning loneliness and being a burden are supported by a study among elderly people living in German nursing homes from Erichsen et al.. An important aspect for the elderly patients in the study of Erichsen et al. was the feeling of being connected to family. Elderly patients feared to burden their family with their own troubles and worries as well as they feared that there is limited interest in their concerns [16], like practitioners also expressed it in our study. The authors also declared that it remains open how to support these unmet needs [16]. Practitioners in our study tried to ask and connect with their patients in different situations, as well as tried to involve relatives in their care.

The challenges of practitioners concerning lack of time and resources to adequately supply care for patients with multimorbidity are in line with the findings of a recent review [17]. Data makes clear that GP views are framed by specific national or regional policy levers impacting on the level of their own practice and their patient care. Practitioners in our study wished for more governmental support concerning mobility and public transport, respectively for elderly people as well as a in prevention of chronic diseases. Patients wished for regular stays in rehabilitation centres, but prescription is temporally bonded to laws [18], disregard wishes and need of patients.

### Need for further support

Only a minority of patients share issues about loneliness with their GP, even though the wish for more support to increase social activity is high [11]. A qualitative study found that primary care physicians had difficulty in defining their tasks concerning loneliness of patients and experienced a lack of therapeutic options [19]. Nevertheless, reviews have pointed out the effectiveness of interventions to reduce loneliness in elderly people [20–22]. The successful interventions were offering social activity and/or support within a group format as well as those who included older people as active participants. It may be important for primary care practices to address loneliness in elderly patients with multimorbidity and to identify those who need support. Furthermore, social events like proposed from the practitioners where elderly people gather for a meal and socialise should be promoted. Practical implementation could be realized via social non- governmental organizations like “Malteser Hilfsdienst” or “Deutsches Rotes Kreuz”, which could organize transport services, as they are doing food delivery for elderly people, currently. Primary care practices could cooperate with them to activate elderly patients to participate.

An analysis of successful urban and transport planning for the aging population found four groups of elderly people concerning social activity: “a group that mainly socializes at home, a group that mainly socializes at a community centre, a group that is more likely to socialize at public ‘third’ places” as well as non-socializers [23]. The authors pointed out that local policy makers should strive to maintain community centre to maintain social participation of the elderly [23]. For the non-socializers providing safe and accessible public transport and walkable neighbourhoods to stimulate their mobility, could promote social participation and health of elderly. These were also proposals from the practitioners of our study. In combination with a health lecture, it could be stimulation and motivation, respectively simultaneously. However, these are in fact current fields of policy and not of health care professionals. Nevertheless, its relevant for health care systems, since a lack of social participation may result in social isolation and loneliness, which may cause a reduction in physical activity and both mental and physical health [10, 24].

In Germany, disease management programs (DMPs) for individuals with diabetes, coronary heart disease and asthma/chronic obstructive pulmonary disease (COPD) are run by primary care practices. The core intervention of DMPs are regular appointments every 3–6 months during which a treatment protocol has to be completed. These serve for many patients as control and space where they can discuss their needs. Furthermore, one part of the DMPs are that all patients should participate in training sessions which focus on disease-specific knowledge and skills (e.g., correct use of inhaler devices for COPD or nutrition counselling for diabetes) [25]. As patients in our study expressed needs for possibilities, they could do in addition for their well-being it could be helpful to include self-care activities (e.g., relaxation exercises or balance exercises), which focus on general well-being, in DMPs. Nevertheless, in a qualitative study concerning health professionals’ views of success in their work with people with multimorbidity, they identified a wide range of interlinked aspects relating to health, wellbeing, and quality of life, how well patients manage and relationship between professionals and patients [26]. This reflects the complexity of multimorbidity care as well as the practitioners’ narrative in our study to how individual care is and that every patient needs another approach to participate and being motivated in self-management.

### Implication for practice and policy

To meet the described aspects of patients with multimorbidity practitioners could use the biopsychosocial model of the International Classification of Functioning, Disability and Health (ICF) to involve role and emotional management in primary care [27]. The model considers not only physical but also psychological and social factors and was developed by the WHO to gather information to health restrictions exactly. In Germany prescription of rehabilitation services is based on the principles of the ICF [27]. Current projects concerning the use of ICF in Germany are also mainly in the field of rehabilitation or for children with chronic disease or disabilities [28]. Whereby, research is still needed on the concrete use of ICF-based instruments and its intended and unintended effects [29]. However, especially in the treatment of elderly people with multimorbidity in primary care, the inclusion of ICF principles may be a great opportunity, since activation, social participation as well as personal environment are crucial factors in their diseases and treatment. Furthermore, the proposal of a social medical service in every municipality as well as an interprofessional approach could facilitate to meet the challenges of lack time and resources of practitioners and more involvement of role and emotional management for patients with multimorbidity. Future research could address if social events like proposed from a physician are wished and accepted by patients as well as the practicability should be evaluated. Moreover, how to unmet needs of role and emotional management in patients with multimorbidity should be explored. Furthermore, it should be examined if and how social medicine as well as the ICF model could be implemented in primary care practices and community resources.

The findings of this study imply a need for ongoing training in patient-centred communication, whereas loneliness and social participation should be addressed. It should also be a priority to develop guidelines for managing multimorbidity that focus on personal continuity, individualized consultation length, and multidisciplinary care. Self-management support should not only help control disease but also empower patients and help to cope with their role and emotions. Furthermore, domestic and community resources have to be extended and access for elderly people should be facilitated. Findings also imply a need for a comprehensive connection between primary care practices and community organisations.

### Strengths and limitations

Due to the global COVID-19 pandemic fewer interviews then planned were conducted. Most patients wanted to do the interview in person and not over the telephone, whereas practitioners declared higher workload without time to participate in an interview. Whereas the inclusion of different views (physicians, practice assistants and patients) increases data variation, the patient sample is heterogeneous concerning age and diseases. Therefore, transferability and comparability with other samples may not possible. Although respondents were mostly outspoken about their experiences, social desirability cannot be precluded. This analysis was guided by an appropriate methodological strategy to minimize research bias and reduce the risk of losing relevant content. Another strength is the randomization in sampling. In addition, the analysis was done by the first author individually and was compared during several meetings with the second author to ensure consistent coding as well as the coding system was discussed with the third author.

## Conclusion

This study presents patients’ and practitioners’ perspectives on status, challenges, and needs of self-management support. Patients and practitioners perceived mainly challenges in loneliness, changing habits or coping with the disease. Patients wished for further support from their primary care physicians whereas practitioners suggested needs in community resources. Challenges regarding self-management of disease may be addressed by harnessing social support and community initiatives as well as by creating a connection to primary care.

## Data Availability

The datasets generated and/or analysed during the current study are not publicly available due to European Data Protection Law but are available from the corresponding author on reasonable request.

## Abbreviations

COPD: Chronic obstructive pulmonary disease
DMP: Disease management program
ICF: International Classification of Functioning, Disability and Health
MULTIqual: Mixed methods study named Development and Validation of Quality Indicators for Multimorbidity
Practitioners: Primary care physicians and practice assistants
VERAH: Care Assistants in General Practice (German: Versorgungsassistentin in der Hausarztpraxis)
